# A tropical Atlantic species of *Melibe* Rang, 1829 (Mollusca, Nudibranchia, Tethyiidae)

**DOI:** 10.3897/zookeys.316.5452

**Published:** 2013-07-11

**Authors:** Erika Espinoza, Anne DuPont, Ángel Valdés

**Affiliations:** 1 Department of Biological Sciences, California State Polytechnic University, 3801 West Temple Avenue, Pomona, California 91768, USA; 2 4070 NW 7th Lane, Delray Beach, Florida 33445, USA

**Keywords:** new species, molecular taxonomy, anatomy, Opistobranchia, western Atlantic

## Abstract

A new species of *Melibe* is described based on two specimens collected in Florida. This new species is well differentiated morphologically and genetically from other species of *Melibe* studied to date. The four residue deletions in the cytochrome c oxidase subunit 1 protein found in all previously sequenced tropical species of *Melibe* sequenced (and *Melibe rosea*) are also present in this new species. These deletions do not appear to affect important structural components of this protein but might have fitness implications. This paper provides the first confirmed record of *Melibe* in the tropical western Atlantic Ocean.

## Introduction

*Melibe* Rang, 1829 (family Tethyiidae Rafinesque, 1815) is an unusual genus of cladobranch nudibranchs that feed by expanding a large oral hood fringed with sensory tentacles to capture small crustaceans. The digestive morphology of this group is largely modified, all species lack a radula and have a circularized stomach (Gosliner and Smith 2003).

Gosliner and Smith (2003) reviewed the systematics of *Melibe* and concluded that there are 14 valid species and three uncertain species. According to Gosliner and Smith (2003) *Melibe* includes a mixture of temperate and tropical species. Temperate species include *Melibe australis* (Angas, 1864) and *Melibe maugeana* Burn, 1960 from southern Australia, *Melibe leonina* (Gould, 1852) from California, and *Melibe rosea* Rang, 1929 and *Melibe liltvedi* Gosliner, 1987 from South Africa. Most tropical species have widespread ranges in the tropical Indo-Pacific including *Melibe viridis* (Kelaart, 1858), *Melibe pilosa* Pease, 1860, *Melibe papillosa* (de Filippi, 1867), *Melibe bucephala* Bergh, 1902, *Melibe engeli* Risbec, 1937, *Melibe megaceras* Gosliner, 1987, and *Melibe digitata* Gosliner & Smith, 2003. Only *Melibe minuta* Gosliner & Smith, 2003 and *Melibe tuberculata* Gosliner & Smith, 2003 appear to have restricted ranges (in Okinawa and the Philippines, respectively). In a recent paper Gosliner and Pola (2012) described two additional new species, *Melibe coralophilia* Gosliner & Pola, 2012 and *Melibe colemani* Gosliner & Pola, 2012, both from the tropical Indo-Pacific and provided the first molecular phylogeny for this group conforming the sister-group relationship of *Melibe* with *Tethys* Linnaeus, 1767, and the monophyly of both *Melibe* and Tethyiidae.

*Melibe* is also unusual biogeographically as it appears to be completely absent from the tropical Eastern Pacific and is poorly represented in the Atlantic Ocean. The only two confirmed records from the Atlantic are the two South African species *Melibe rosea* and *Melibe liltvedi*, which are found on the Atlantic side of the Cape Peninsula. *Melibe viridis* has been reported from the Mediterranean – originally as *Melibe fimbriata* (Alder & Hancock, 1864), but is considered a non-native species (Thompson and Crampton 1984). The only record of *Melibe* in the tropical Atlantic Ocean is a photograph of a potentially undescribed species found in Guanaja, Honduras, Caribbean Sea (Valdés et al. 2006), but this record has never been confirmed with the examination of specimens.

In this paper we describe the first species of *Melibe* from the tropical Atlantic Ocean based on two specimens recently collected in Florida. Molecular and morphological data obtained from the two specimens are compared with other congeners.

## Methods

### Source of specimens and morphological examination

Two specimens were collected by SCUBA diving in Lake Worth Lagoon Florida, photographed alive, and preserved in pharmacy grade ethyl alcohol. Once in the laboratory they were transferred to ethanol 95%. All the specimens examined are deposited at the Natural History Museum of Los Angeles County, USA (abbreviated LACM).

The anterior portion of the digestive system and the reproductive system were dissected and drawn under a dissecting microscope with a camera lucida attachment. The stomach was also dissected to expose the stomach plates. The buccal mass was dissolved in a NaOH 10% solution to isolate the jaws. The jaws and the stomach were rinsed in distilled water, dried, mounted on a stub, and sputter coated for examination under a scanning electron microscope (SEM) Hitachi S-3000N.

### DNA extraction, PCR amplification and sequencing

DNA extraction was performed using a hot Chelex® protocol. Approximately 1-3 mg of the foot was cut into fine pieces for extraction. For the Chelex® extraction, the foot tissue was rinsed and rehydrated using 1.0 mL TE buffer (10 mM Tris, 1 mM EDTA, pH 8.0) for 20 minutes. A 10% (w/v) Chelex® 100 (100-200 mesh, sodium form, Bio-Rad) solution was prepared using TE buffer. After rehydration, the tissue mixture was then centrifuged, 975.00 μL of the supernatant was removed, and 175.00 μL of the Chelex® solution was added. Samples were then heated in a 56°C water bath for 20 minutes, heated in a 100°C heating block for 8 minutes, and the supernatant was used for PCR.

Histone-3 universal primers (H3 AF 5’-ATGGCTCGTACCAAGCAGACGGC-3’, H3 AR 5’-ATATCCTTGGGCATGATGGTGAC-3’ developed by Colgan et al. 1998), 16S rRNA universal primers (16S ar-L 5’-CGCCTGTTTATCAAAAACAT-3’, 16S br-H 5’- CCGGTCTGAACTCAGATCACGT-3’ developed by Palumbi 1996), and CO1 universal primers (LCO1490 5’-GGTCAACAAATCATAAAGATATTGG-3’, HCO2198 5’-TAAACTTCAGGGTGACCAAAAAATCA-3’ developed by Folmer et al. 1994) were used to amplify the regions of interest for all specimens.

The master mix was prepared using 34.75 μL H2O, 5.00 μL Buffer B (ExACTGene, Fisher Scientific), 5.00 μL 25 mM MgCl2, 1.00 μL 40mM dNTPs, 1.00 μL 10mM primer 1, 1.00 μL primer 2, 0.25 μL 5 mg/mL Taq, and 2.00 μL of extracted DNA. Reaction conditions for H3 (universal) and 16S (universal) were as follows: lid heated to 105°C and initial denaturation of 94°C for 2 min, 35 cycles of 94°C for 30 s, 50°C for 30 s, and 72°C for 1 min, followed by a final elongation of 72°C for 7 min. Reaction conditions for CO1 (universal) were as follows: lid heated to 105°C and initial denaturation of 95°C for 3 min, 35 cycles of 94°C for 45 s, 45°C for 45 s, and 72°C for 2 min, followed by a final elongation step of 72°C for 10 min. PCR products yielding bands of appropriate size were purified using the Montage PCR Cleanup Kit (Millipore). Cleaned PCR samples were quantified using a NanoDrop 1000 Spectrophotometer (Thermo Scientific). Each primer was diluted to 4.0 pmol/μL to send out for sequencing with the PCR products. PCR products were diluted to 6.0, 7.5, and 11.5ng/μL for H3, 16S, and CO1 respectively. Samples were sequenced at Eton Bioscience, Inc. (San Diego, CA).

Sequences were deposited in GenBank (http://www.ncbi.nlm.nih.gov/genbank/) with the accession numbers KC992314 for CO1, KC992313 for 16S, and KC992315 for H3. Sequences of other species of *Melibe* and *Tethys fimbria* Linnaeus, 1767 were downloaded from GenBank (Table 1) and included in the phylogenetic analysis. Sequences for each gene were assembled and edited using GENEIOUS Pro 4.7.4 (Drummond et al. 2010). GENEIOUS Pro 4.7.4 was also used to extract the consensus sequence between the primer regions, construct the alignment for each gene using the default parameters, concatenate the alignments, translate the CO1 sequences intro protein sequences and align the protein sequences. The sequences were not trimmed after alignment. A total of 328 bp for H3, 453 bp for 16S, and 657 bp for CO1 including gaps were used for the phylogenetic analyses.

**Table 1.** Specimens sequenced, including locality information, collection voucher numbers and GenBank accession numbers.<br/>

**Species**	**Voucher**	**Locality**	**GenBank accession numbers**		
			**CO1**	**16S**	**H3**
*Tethys fimbria*	-	-	AY345035	AY345035	EF133468
*Melibe leonina*	LACM174849	California, USA	GQ292059	GU339202	-
*Melibe digitata*	CASIZ175724	Philippines	JX306069	JX306061	JX306076
*Melibe digitata*	CASIZ177478	Philippines	HM162699	HM162617	HM162523
*Melibe viridis*	CASIZ176981	Mozambique	JX306075	JX306068	JX306083
*Melibe viridis*	CASIZ177524	Philippines	HM162700	HM162618	HM162524
*Melibe rosea*	CASIZ175734	South Africa	JX306070	JX306063	JX306078
*Melibe rosea*	CASIZ176355	South Africa	JX306071	JX306064	JX306079
*Melibe rosea*	CASIZ176367	South Africa	JX306073	JX306066	JX306081
*Melibe rosea*	CASIZ176356	South Africa	JX306072	JX306065	JX306080
*Melibe rosea*	CASIZ176392	South Africa	HM162701	HM162620	HM162526
*Melibe engeli*	CASIZ177625	Philippines	-	HM162619	HM162525
*Melibe engeli*	CASIZ177757	Philippines	-	JX306062	JX306077
*Melibe arianeae*	LACM3259	Florida, USA	KC992314	KC992313	KC992315

### Phylogenetic analyses

To assess whether H3, 16S, and CO1 have significantly conflicting signals the incongruence length difference (ILD) test (Mickevich and Farris 1981, Farris et al. 1994), implemented in PAUP*4.0 as the partition homogeneity test (Swofford 2002), was conducted for all genes combined. The levels of saturation for each gene and for the first and second versus third codon positions of CO1 and H3 were investigated using the substitution saturation test developed by Xia et al. (2003) and Xia and Lemey (2009) implemented in the program DAMBE 4.0 (Xia and Xie 2001).

The Akaike information criterion (Akaike 1974) was executed in MRMODELTEST 2.3 (Nylander 2004) to determine the best-fit model of evolution. Bayesian analyses were executed in MRBAYES 3.2.1 (Huelsenbeck and Ronquist 2001), partitioned by gene (unlinked), with *Tethys fimbria* as the outgroup. Outgroup selection was based on the sister relationship between *Tethys* and *Melibe* (Gosliner and Pola 2012). The Markov chain Monte Carlo analysis was run with two runs of six chains for ten million generations, with sampling every 100 generations. Effective sample sizes and convergence of runs were assessed using TRACER 1.4.1 (Rambaut and Drummond 2007). The default 25% burn-in was applied before constructing majority-rule consensus tree/s.

## Results

The saturation analysis showed insignificant levels of saturation for all three genes, CO1: Iss (0.4398) < Iss.c (0.7384), *P* = 0.000; 16S: Iss (0.6502) < Iss.c (0.7087), *P* = 0.007; H3: Iss (0.5591) < Iss.c (0.7193), *P* = 0.000. The ILD test showed NS conflicting signals between the genes combined: CO1 vs. H3 (*P* = 0.99), and 16S vs. H3 (*P* = 0.08), except CO1 vs. 16S (*P*= 0.001). MRMODELTEST 2.3 selected the models GTR+I+G for CO1 and 16S and GTR+I for H3 (CO1 γ shape = 0.34, proportion of invariant sites = 0.26; 16S γ shape = 0.84, proportion of invariant sites = 0.24; H3 proportion of invariant sites = 0.81).

**Figure 1.**
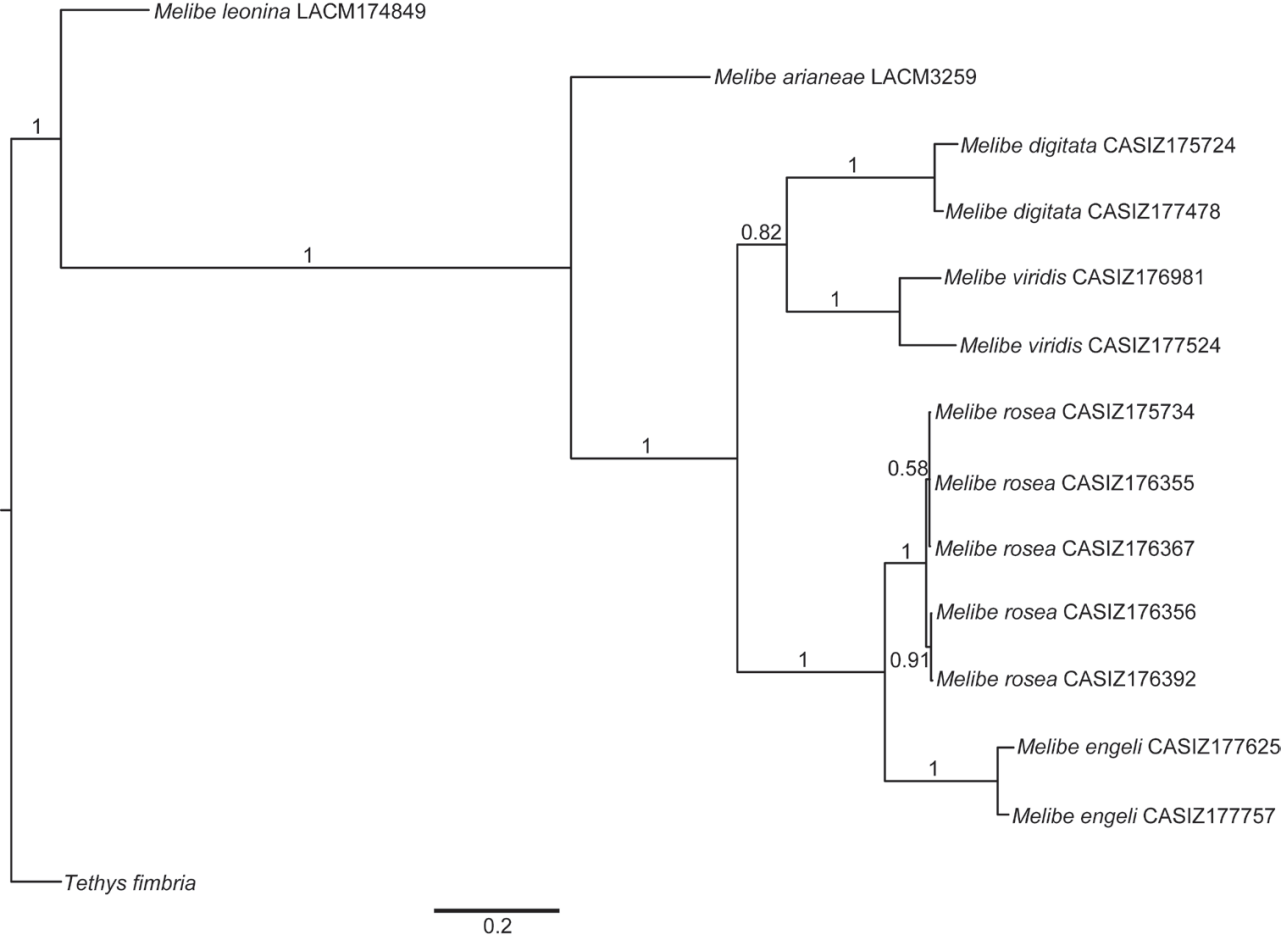
Bayesian consensus tree of *Melibe* including museum voucher numbers and posterior probabilities. Abbreviations: CASIZ, California Academy of Sciences, Invertebrate Zoology; LACM, Natural History Museum of Los Angeles County.

The combined analysis of the three genes (H3, 16S, and CO1) produced a consensus Bayesian tree in which the monophyly of *Melibe* is well supported, posterior probability (pp) = 1 (Fig. 1). Within *Melibe*, the temperate species *Melibe leonina* is the most basal, however this result must be taken cautiously as several other species were not included in the analysis. For the rest of the species analyzed, *Melibe arianeae* sp. n. is sister to the tropical Indo-Pacific and South African taxa (pp = 1), which includes the Mediterranean non-native *Melibe viridis* as well as *Melibe engeli*, the species morphologically more similar to *Melibe arianeae*. All the species with more than one specimen included in the analysis (*Melibe digitata*, *Melibe viridis*, *Melibe rosea* and *Melibe engeli*) are monophyletic and well supported (pp = 1).

When aligned with other species of opisthobranchs including *Tethys*, the CO1 sequence of *Melibe arianeae* as well as those of other tropical species of *Melibe* available in GenBank show 4 codon deletions. These codons are in positions 87-89, 352-354, 470-472, and 473-475 (2) of the partial sequence alignment.

### Systematics

#### Melibe arianeae sp. n.

urn:lsid:zoobank.org:act:B9B242B1-9440-4AC3-88D9-A7260986172E

http://species-id.net/wiki/Melibe_arianeae

Figs 2 – 4

Melibe sp. Valdés et al., 2006: 234-235.

##### Type-locality.

Lake Worth Lagoon, Palm Beach County, Florida (26.782781, -80.04468), 3 m depth, 6 mm preserved length, April 6, 2013, A. Dimitris leg.

##### Type-specimens.

Holotype preserved in ethanol 95%, dissected but no organs removed (LACM 3258). Paratype preserved in ethanol 95%, dissected, reproductive system in the same vial, stomach on a SEM stub (LACM 3259).

##### Description.

**Figure 2.**
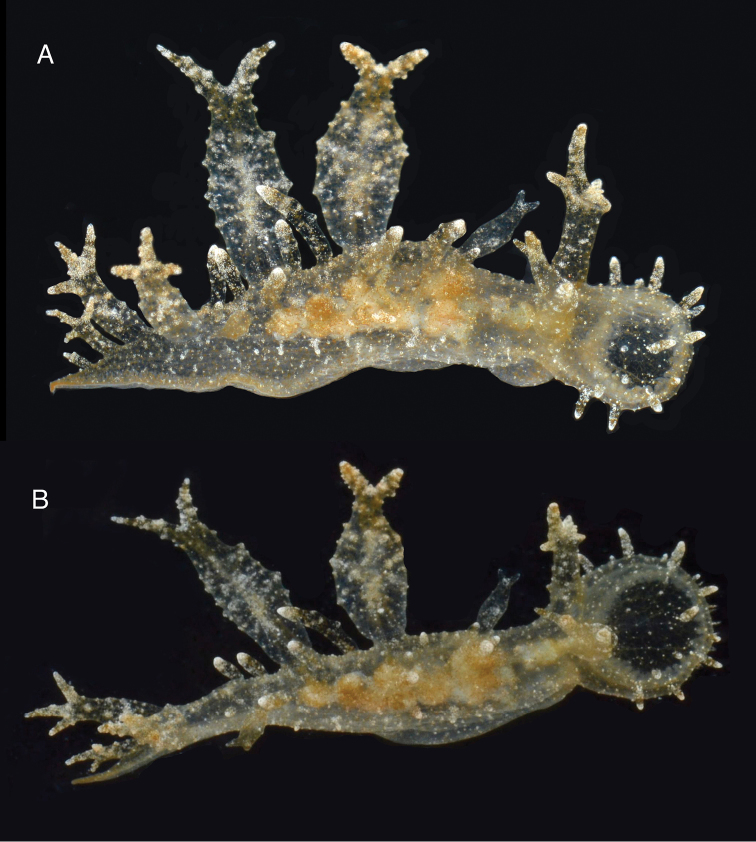
Two views of the holotype of *Melibe arianeae* sp. n. (LACM 3258). A. Dorsolateral view showing the right side of the animal. B. Dorsal view showing the oral hood border through the semi-transparent skin.

The living animals are nearly transparent, with numerous orange flecks and opaque white blotches all over its surface, and orange-brownish colored internal organs (Fig. 2). The body is limaciform and elongate, somewhat compressed anterolaterally, tapering posteriorly into a long, conical posterior end of the foot. The entire body surface, including cerata and rhinophoral sheaths are covered by numerous minute, opaque white tubercles. In the center of the dorsum of the holotype there are several (8) transparent tentacular papillae of different sizes, also covered with small white tubercles and having opaque white apices. The foot sole is wider anteriorly, it is covered with orange flecks and opaque white blotches as the dorsal surface, but it also has a faint white rim. The circular oral hood is small compared to the rest of the body. The margin of the hood is entire (with no indentations) and bears two rows of elongate papillae. There are papillae on the dorsal surface of the hood, generally resembling those on the body surface, and more concentrated towards the anterior margin. The rhinophores emerge from the posterior end of the oral hood. The rhinophores have 3–4 perfoliations.The rhinophoral sheaths are somewhat inflated and cylindrical, lacking a leaf-like posterior process. The sheaths have 2–3 posterior papillae. The cerata are inflated, oval, completely covered with small tubercles that give it a broadly warty look. Their distal ends of the cerata are either simple, bifurcate or trifurcate, independently of their size. The cerata are transparent, and the branches of the digestive gland within them are visible as a brownish axis. There are seven cerata alternating on each side of the dorsal midline of the holotype. The anus is located dorsol-lateraly, midway between the first and second anterior cerata. The position of the nephroproct could not be determined. The gonopore is lateral, anterior to the anteriormost right ceras. There are no papillae associated with the gonopore.

**Figure 3.**
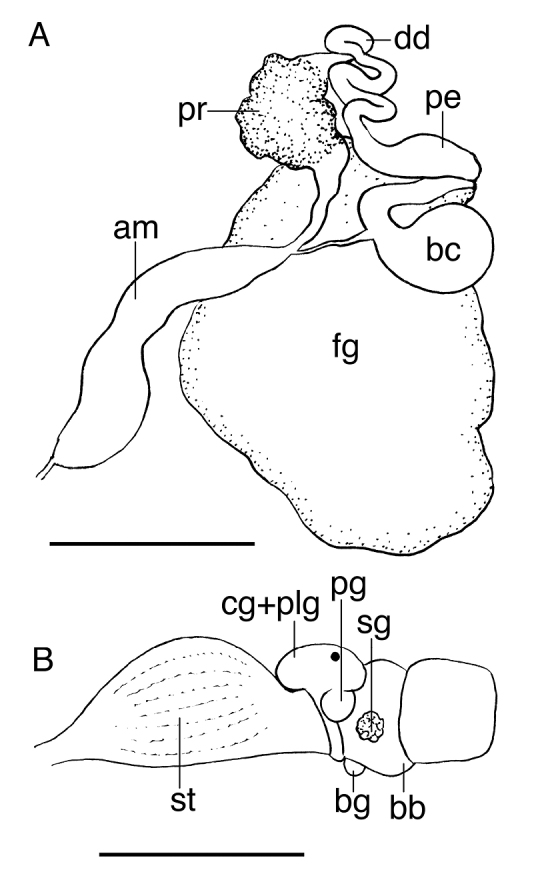
**A** Reproductive system of the paratype of *Melibe arianeae* sp. n. (LACM 3259) **B** Anterior portion of the digestive system of the holotype of *Melibe arianeae* sp. n. (LACM 3258). Scale bars = 1mm. Abbreviations: am, ampulla; bb, buccal bulb; bc, bursa copulatrix; bg, buccal ganglion; cg, cerebral ganglion; dd, deferent duct; fg, female gland complex; pe, penis; plg, pleural ganglion; pg, pedal ganglion; pr, prostate; sg, salivary gland; st, stomach.

**Figure 4.**
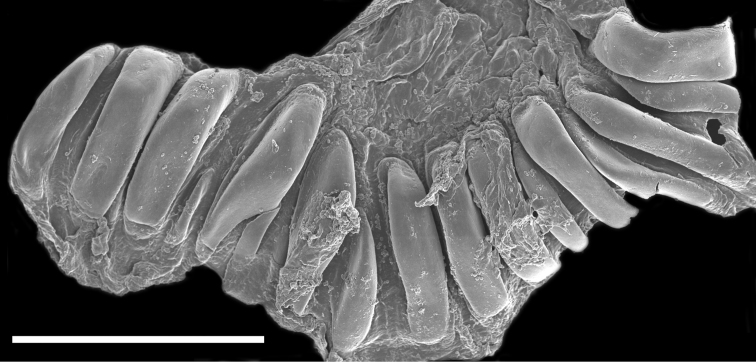
Scanning electron micrograph of the dissected stomach of the paratype of *Melibe arianeae* sp. n. (LACM 3259) showing the stomach plates. Scale bar = 500 µm.

The buccal mass is devoid of a radula but contains a pair of simple, chitinous jaws. The jaws (not illustrated) have smooth borders and lack denticles on the masticatory border. The short esophagus emerges from the posterior end of the buccal mass and expands into a muscular stomach (Fig. 3B). Two small salivary glands are located laterally, one on each side of the buccal mass. The posterior portion of the stomach contains 18 elongate, thick and robust chitinous plates of various sizes (Fig. 4). The reproductive system is triaulic and contains a series of four spherical, well-separated ovotestis bodies, connected to a large ampulla. The ampulla connects into the female gland complex (Fig. 3A) near the point where the prostate emerges. The prostate is a short and wide glandular structure connected to a long, and convoluted deferent duct that expands distally into the penial sac. The vagina is short and wide and connects directly into a large bursa copulatrix. The narrow and straight uterine duct connects to the female gland complex. A serial seminal receptacle (present in other species of *Melibe*) was not observed. The central nervous system (Fig. 3B) is located above the esophagus and contains a fused pair of cerebral and pleural ganglia, as well as a pair of pedal ganglia. The buccal ganglia are located at the proximal end of the buccal mass.

##### Etymology.

This species is named for Ariane Dimitris, amateur naturalist and passionate sea slug enthusiast, who collected the specimens here examined.

## Discussion

*Melibe arianeae* is well differentiated morphologically relative to other congeners (discussed below). Molecular evidence is provided to support the placement of this new species in *Melibe* and to compare it to other species for which sequence data are available. There are large gaps in the molecular coverage of *Melibe*, thus comparison to all other species is not possible at this point. However, the phylogenetic tree here presented shows that *Melibe arianeae* is sister to a well-supported clade containing some of the most morphologically similar species to *Melibe arianeae*.

*Melibe arianeae* is externally most similar to *Melibe engeli*, originally described from New Caledonia and recently reported from Japan, the Hawaiian Islands and the Philippines (Gosliner and Smith 2003). Both species have a nearly transparent body with large cerata covered with conical tubercles and having bifurcated or trifurcated tips, as well as a large oral hood with two rows of papillae. Distinguishable characteristics include the absence of leaf-like posterior processes on the rhinophoral sheaths of *Melibe arianeae* and the lack of a vestibular gland in this species, both present in *Melibe engeli* (Gosliner and Smith 2003). The body papillae of *Melibe engeli* are more conical than those of *Melibe arianeae* and the cerata of *Melibe engeli* are covered with large papillae giving them a serrate appearance (Gosliner et al. 2008); these are absent in *Melibe arianeae*. Additionally, *Melibe engeli* is much larger, reaching up to 45 mm in length (Gosliner et al. 2008), whereas all specimens of *Melibe arianeae* are smaller than 15 mm. Additionally, *Melibe engeli* and *Melibe arianeae* are genetically distinct, with a pairwise identity of 71.4% in 16S and 96% in H3 (there are no CO1 sequences available for *Melibe engeli*). For comparison, within species pairwise identity values in *Melibe* range between 96.2–99.3% for 16S and between 99.3–100% for H3.

All other species of *Melibe* examined and reviewed by Gosliner and Smith (2003) and Gosliner and Pola (2012) are externally easily distinguishable from *Melibe arianeae*. The California species *Melibe leonina* has flat, smooth, leaf-like cerata and a large oral hood with comparatively small rhinophores. The Australian species *Melibe australis* and *Melibe maugeana* are also easily distinguishable; *Melibe australis* has short, densely papillate flask-like cerata, and a round oral hood with a thick margin, whereas *Melibe maugeana* has a very large oral hood and few, long and cylindrical or fusiform cerata. The other two species found in the Atlantic, the South African *Melibe rosea* and *Melibe liltvedi*, are also clearly different; *Melibe rosea* has a pinkish general color, a large oral hood, and irregular and small club-shaped certata, and *Melibe liltvedi* has a comparatively small hood and very characteristic club-shaped, very large cerata. The tropical Indo-Pacific species exhibit a remarkable morphological diversity that makes most species easily identifiable. All the species are illustrated in Gosliner et al. (2008) based on live animals and here compared to *Melibe arianeae*. *Melibe megaceras* is a very distinctive species with very elongate and bifurcate cerata and a relatively small oral hood. *Melibe digitata* has long cerata with the apices densely covered with long papillae. *Melibe minuta* has long, highly ramified cerata ending in multifid acutely tapering apices. *Melibe tuberculata* is easily identifiable because of the presence of large, stalked tubercles on the cerata. *Melibe papillosa* and *Melibe pilosa* are very similar externally, both have a large oral hood and apically flattened cerata with a regular wedge shaped margin, bearing a few thin papillae. *Melibe bucephala* is a large species with an incised oral hood, the body covered with papillae and the cerata with large apical digitations. *Melibe coralophilia* resembles a live coral head, and it is densely covered with tubercles that form a mid-dorsal crest and cover the surface of the cerata. *Melibe colemani* is a transparent species with a series of white interconnecting digestive gland ducts visible throughout the body. *Melibe viridis*, which has been reported from the Mediterranean Sea, is distinguishable from *Melibe arianeae* by having a large oral hood and flattened, saccate, oval to cylindrical cerata with tubercular and papillate surfaces.

The specimen of *Melibe* sp. illustrated by Valdés et al. (2006) from Honduras probably is *Melibe arianeae* as it shares a similar external morphology, but this needs to be confirmed with examination of specimens. If this record is confirmed the known range of *Melibe arianeae* includes Florida and Honduras.

One of the most intriguing aspects of the genetics of *Melibe* is the presence of four deletions in the sequence of the cytochrome c oxidase subunit 1protein in tropical congeners and *Melibe rosea* from South Africa. A protein alignment revealed that these deletions resulted in the loss of 4 residues in the cytochrome c oxidase subunit 1 protein. The structure of the cytochrome c oxidase of *Paracoccus denitrificans* was reconstructed by Iwata et al. (1995) who found that the subunit 1 contains 12 membrane-spanning, primarily helical segments and binds to haem *a* and the haem *a_3_*-copper B binuclear center where molecular oxygen is reduced. The alignment of the *Melibe arianeae* cytochrome c oxidase subunit 1 sequence with the annotated sequence of *Paracoccus denitrificans* based on the structural data collected by Iwata et al. (1995), shows that the residue deletions are located at the very beginning of helix II and in between helical segments III–IV and IV–V. These deletions do not seem to be affecting the shape of important structural elements, thus their functional implications might be limited. However, the fact that they are only present in tropical species of *Melibe* and the South African species *Melibe rosea* among all nudibranchs sequenced to date (including the temperate species *Melibe leonina* and the closely relatedMediterranean species *Tethys fimbria*), and that some of them are located in highly conserved regions, suggest that they could have important fitness effects for the respiratory electron transport chain of mitochondria.

## Conclusion

Molecular and morphological evidence confirmed that the specimens from Florida here examined belong to *Melibe* and therefore this paper is the first confirmed record of this group in the tropical western Atlantic Ocean. Morphological examinations also confirmed that these specimens constitute an undescribed species, which is morphologically distinct from other species of *Melibe* described to date. Additionally, these specimens are genetically distinct from other species of *Melibe* so far sequenced. Our knowledge of the phylogeny of *Melibe* is spotty, as many species have not been sequenced yet, thus few conclusions can be drawn from the Bayesian consensus tree. However, it clear that the tropical Indo-Pacific species studied so far form a monophyletic group. The presence of four deletions in the sequence of the cytochrome c oxidase subunit 1 protein in some *Melibe* could have important implications to understand protein function and selection on mitochondrial genes.

## Supplementary Material

XML Treatment for Melibe arianeae
